# Usefulness of hemoglobin examination in gingival crevicular fluid during supportive periodontal therapy to diagnose the pre-symptomatic state in periodontal disease

**DOI:** 10.1007/s00784-020-03396-0

**Published:** 2020-06-15

**Authors:** Hiroshi Ito, Yukihiro Numabe, Shuichi Hashimoto, Sunao Uehara, Ya-Hsin Wu, Tomohisa Ogawa

**Affiliations:** 1 Department of Periodontology, School of Life Dentistry at Tokyo, The Nippon Dental University, Tokyo, Japan; 2 The Nippon Dental University, Tokyo, Japan; 3 General Dentistry, The Nippon Dental University Hospital, Tokyo, Japan

**Keywords:** Gingival crevicular fluid, Periodontitis, Hemoglobin, Supportive periodontal therapy

## Abstract

**Objectives:**

The absence of bleeding on probing (BOP) is a good predictor of disease stability. This study investigated whether detection of hemoglobin (Hb) in gingival crevicular fluid (GCF) indicates minute signs of periodontal disease, even in BOP (−) cases.

**Materials and methods:**

GCF was collected from gingival sulci of 152 sound maxillary and mandibular teeth from 76 patients who had entered supportive periodontal therapy (SPT) using the split-mouth design. As clinical parameters, plaque index, GCF amount, gingival index, probing depth (PD), clinical attachment level, BOP, and alveolar bone resorption ratio were then recorded. As biochemical parameters, Hb amount, alkaline phosphatase (ALP) activity, and protein amount in GCF were measured. Periodontal conditions of diseased sites (PD ≥ 4 mm, BOP (+)) and healthy sites (PD ≤ 4 mm, BOP (−)) were further classified into two groups using the Hb cutoff value determined by PD and BOP and analyzed.

**Results:**

Despite being healthy, ALP activity and protein amount in sulci of the group with Hb level greater than the cutoff value were significantly higher than those in the group with Hb level less than the cutoff value (*P* < 0.01).

**Conclusions:**

This study indicates that Hb examination is a promising candidate marker of pre-symptomatic periodontal disease because Hb presence in GCF suggests slight tissue damage, even in healthy sites defined as BOP (−).

**Clinical relevance:**

Hb examination of GCF is a powerful diagnostic tool for pre-symptomatic diagnosis of periodontal disease.

## Introduction

Important items during the period of supportive periodontal therapy (SPT) are the accurate diagnosis of periodontitis and prompt response corresponding to symptoms in order to prevent the recurrence of periodontal disease [1, 2]. Both probing depth (PD) measurement and bleeding on probing (BOP) examination are presently recognized worldwide as extremely effective parameters for assessing periodontal disease and recurrence [3–5]. However, these measurements and examinations are not only inspections requiring delicate skills, but also tests in which the burden on the patient increases due to pain associated with the examination according to the condition of deteriorated periodontal disease [6]. Furthermore, the BOP test has a higher negative predictive value than positive predictive value [7]. Therefore, we focused on gingival crevicular fluid (GCF), which can be painlessly sampled, as a candidate test item to supplement PD measurement and BOP examination in the SPT phase and analyzed its components [8–10].

BOP examination visually judges the presence or absence of bleeding after PD measurement and evaluates periodontal tissue condition [11, 12]. In contrast, we hypothesized that the detection of hemoglobin (Hb), which is already present in GCF by invisible inflammatory bleeding, is effective for pre-disease diagnosis of periodontal disease [9, 10]. Therefore, we measured the Hb amount in GCF, which is an indicator of bleeding in the periodontal pocket, and analyzed the correlation between Hb amount and other periodontal tissue examinations. We reported that Hb amount in GCF collected from 401 pockets in 184 SPT patients correlates well with assessments of clinical parameters (e.g., plaque index (PlI), GCF amount, PD, clinical attachment level (CAL), gingival index (GI), and BOP) for periodontal condition [10]. As a result, we found that Hb examination could be a helpful test item, which complements conventional periodontal tissue inspections [9, 10].

However, it is unclear whether the presence of Hb in GCF could be a marker of periodontal tissue damage. Therefore, when BOP is negative, it is not known whether Hb in GCF is present at the site where there is a possibility of periodontal tissue damage. In particular, we hypothesized that Hb in GCF, which can be expressed in the case of BOP (−), is effective for detecting the pre-symptomatic state in periodontal disease.

In the present study, we used a split-mouth design to eliminate differences between individuals in periodontal tissue examination and increase reliability. GCF was simultaneously collected from both healthy and diseased sites in the same oral cavity in each patient, and the components in GCF were analyzed [13]. We then compared each clinical parameter (PlI, GCF amount, GI, PD, CAL, and alveolar bone resorption (ABR) ratio) and Hb amount, in addition to ALP activity and protein amount as biochemical parameters in GCF that increase with the onset of periodontitis [8, 14–19]. Finally, we checked for correlations between the examinations of conventional clinical and biochemical parameters and the examination of Hb. We believe that the present evaluation of the usefulness of Hb inspection in GCF in periodontal diagnosis confirms that the Hb amount can be used to identify the pre-symptomatic state in periodontal disease.

## Materials and methods

### Experimental design

This study was a cross-sectional study, using a split-mouth design. Patients were randomized into one of two groups. The study was prepared according to the STROBE guidelines.

### Patients

Patients were 76 nonsmokers (39 men, 37 women; mean age 63.4 ± 10.8 years) who were receiving SPT at a periodontal specialty clinic at the Nippon Dental University Hospital (mean SPT period, 5.2 ± 7.1 years). The history of periodontal treatment was 2.4 ± 1.5 years during the period of active periodontal treatment. GCF was collected from 96 patients using the split-mouth design. Among these patients, there were 76 healthy and diseased site pairs. Thus, 20 patients without both healthy and diseased sites were excluded from the analyses.

Specifically, in order to confirm that we can shift the SPT period, we performed the periodontal tissue examination including ABR rate determined by the X-ray image. From the latest periodontal tissue examination including the X-ray image and visual examination of the gingival tissue with marginal gingival position immediately before GCF collection, we predicted healthy and diseased sites and collected GCF randomly. As a result, out of 96 patients, 76 patients had both healthy and diseased sites.

In addition, Table 1 shows the classification of periodontal disease [20] for 76 patients.

**Table 1:** Classification of periodontitis in patients (*n* = 76)

Stage	I				II			III			IV		
Grade	A	B	C		A	B	C	A	B	C	A	B	C
Localized periodontitis	–	–	–		2	13	–	–	1	2	–	–	–
Generalized periodontitis	–	–	–		–	3	1	–	14	5	–	20	15

Inclusion criteria were that the patient had at least 12 remaining natural teeth (number of naturally remaining teeth, 22.4 ± 4.0) and was generally healthy. Exclusion criteria were as follows: (1) patients with diabetes, immune disorders, liver disease, heart disease, or osteoporosis; (2) females who were pregnant or taking birth control pills; (3) patients who had received antimicrobial therapy for the past 3 months; and (4) patients who did not provide consent to participate in this study. Prior to commencing this study, patients received an explanation of the study and were asked to provide written consent.

This study was conducted in accordance with the Helsinki Declaration of 1975, as revised in 2013, with approval from the ethics committee of the Nippon Dental University Hospital (Approval Nos. NDU-T 2017-12 and NDUH-RINRI2018-07).

### GCF collection and preparation

GCF was sampled from a single tooth corresponding to each of the following clinical parameters on both healthy and diseased sites of the same patient. In other words, we compared clinical and biochemical parameters in GCF for both healthy and diseased sites from SPT using the split-mouth design to exclude the possibility of individual differences due to different oral environments.

Criteria for sampling sites were as follows: for healthy sites, PD ≤ 4 mm and BOP (−) and, for diseased sites, PD ≥ 4 mm and BOP (+) [7, 21–23]. Abutment teeth for partial dentures, full crowns, and implants were excluded.

In order to collect GCF, after measuring the PlI [24], the pocket was simply dried using cotton rolls and air, and the overlying plaque was removed as much as possible. Subsequently, a blotting paper strip (PerioPaper®, Oraflow Inc., Plainview, NY, USA) was inserted into the pocket until resistance was felt, and GCF was collected with paper for 30 s. The process for collecting GCF was repeated three times using three paper strips. The amount of GCF collected was measured using a calibrated unit (Periotron® 8000, Oraflow Inc.) and expressed as microliters (μL). The three paper strips used to sample the GCF were immediately soaked in 350 μL of phosphate-buffered saline (PBS), stirred for 5 min, and centrifuged for 5 min at 10,000 rpm. The supernatant was dispensed for biochemical analysis. The recovery rate of all biochemical parameters into the solvent from the paper strips that collected GCF was 98% or greater. If bleeding happened by insertion of the paper strip, the GCF sample was considered to be contaminated with blood and was excluded. Each GCF sample was stored at − 80 °C until further analysis [9, 10, 13].

### Measurement of clinical parameters

All subjects underwent periodontal tissue examination by a specialist periodontist (HI), who is certified by the Japanese Society of Periodontology (Registration No. 147). After calibration, a 95.6% concordance rate (*n* = 12) within 1 mm for PD and CAL measurements between the first and second recording with a 24-h interval was reached.

To prevent blood contamination from the periodontal tissue examination during GCF collection, GCF was collected after PlI measurement. Then, GI [25], PD, CAL, BOP [12], and ABR ratio were assessed after GCF sampling. In the X-ray image used to measure the ABR ratio, the long cone technique was used for standardization. PD and CAL were measured using a pocket probe (Williams Probe, Hu-Friedy Inc., Chicago, IL, USA) with standardized force ranging 20–25 g [4]. PD was measured to the nearest millimeter (mm). Finally, BOP was recorded. To measure the ABR ratio, we used the modified ruler by Schei et al. [26]; the ABR ratio was measured from the tooth root length and alveolar bone height obtained from the transmitted dental X-ray image of each site.

Finally, 152 GCF samples from 76 patients were collected from September 2015 to October 2016 at the Nippon Dental University Hospital and included in subsequent analyses.

### Analysis of biochemical parameters

#### Biochemical parameters included Hb amount, ALP activity, and protein amount

Hb amount in GCF was measured using an Hb detection kit (Check-Line Hemo®, Wakamoto, Tokyo, Japan) using immunochromatography (IC) with a human Hb monoclonal antibody [27, 28]. A 10-μL sample of PBS extraction was added to 40 mL of IC solvent containing bovine serum albumin, polysorbate 20 (Tween 20, Sigma-Aldrich, St. Louis, MO, USA), and sodium acid in neutral PBS, and then, Hb in 50 μL of the solution was developed at 23 °C for 15 min with IC paper. The developed IC paper was then air-dried overnight, and the amount of human Hb monoclonal antibody tinged with red latex in the chromatogram was measured by a densitometer (GS-800 Calibrated Densitometer PC system, Bio-Rad, Tokyo, Japan). The pocket site was defined as positive for Hb at ≥ 1 ng/pocket Hb.

ALP activity in the GCF sample was expressed as μU/pocket using the measurement conforming to the Bessey–Lowry method [14]. The enzyme reaction was performed with p-nitrophenyl phosphate, and resultant p-nitrophenol was measured using a microplate reader at 405 nm.

To measure protein amount in the GCF sample, we utilized the protein assay kit (BCA™ Protein Assay Kit, Thermo Fisher Scientific, Waltham, MA, USA), and values are expressed as μg/pocket.

### Statistical analysis

Clinical and biochemical parameters are expressed as means and standard deviation (SD). All analyses were performed using statistical software (PASW 18.0.0, SPSS, Chicago, IL, USA).

The Kolmogorov–Smirnov normality test was used to determine normality, and none of the clinical and biochemical parameters exhibited normal distribution (*P* < 0.01).

We used the Mann–Whitney *U* test to compare healthy and diseased sites. Correlations between clinical and biochemical parameters were examined using Spearman’s correlation coefficient. In addition, based on criteria for healthy and diseased sites, we prepared a cutoff value for Hb amount using the receiver operating characteristic (ROC) curve and Youden index.

**Fig. 1:**
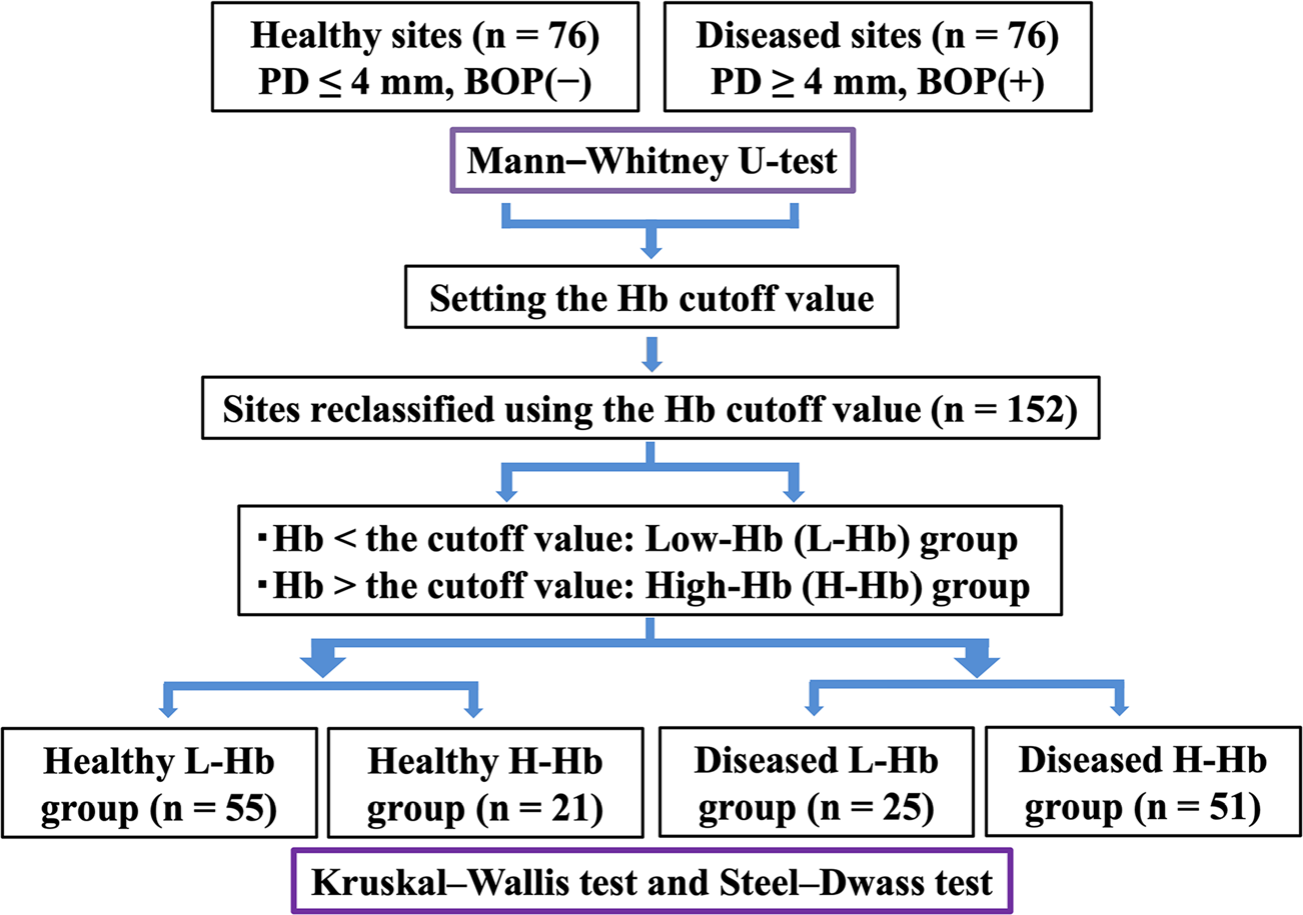
Flowchart of 152 GCF samples from 76 patients

Healthy site and diseased site groups were respectively reclassified into two groups of less than the cutoff value of Hb amount in GCF (low-Hb, L-Hb) and greater than the cutoff value of Hb amount in GCF (high-Hb, H-Hb). Consequently, these gingival sulcus sites were divided into four groups: healthy L-Hb group, healthy H-Hb group, diseased L-Hb group, and diseased H-Hb group corresponding to periodontal disease progression. After Kruskal–Wallis analysis of the four groups, mutually significant differences among the four groups were confirmed with the Steel–Dwass test as a post hoc analysis. Statistical significance was set at *P* < 0.05. The workflow of this study is shown in Fig. 1.

Interquartile range analysis was performed for all clinical and biochemical parameters.

Power calculations indicated a sample size of 64 patients and 128 GCF samples to be necessary (alpha, 0.05; power, 0.8). We thus enrolled 76 patients and collected 152 GCF samples to account for any potential complications (e.g., consent withdraw or insufficient sample volume).

## Results

### Comparison of clinical and biochemical parameters between healthy and diseased sites in the same oral cavity

Table 2 shows clinical parameters (PlI, GCF amount, GI, PD, CAL, BOP, and ABR ratio) and biochemical parameters (Hb amount, ALP activity, and protein amount). All clinical and biochemical parameters were significantly higher in diseased sites (*n* = 76) than in healthy sites (*n* = 76) (*P* < 0.01). In particular, Hb amount in GCF samples from 152 sites ranged 1–204 ng/pocket.

**Table 2:** Comparison between healthy sites and diseased sites

Clinical and biochemical parameters	Sites	Mean ± SD	First quartile	Median	Third quartile
PlI	Healthy sites	0.1 ± 0.4	0.0	0.0	0.0
	Diseased sites	0.6 ± 0.7^***^	0.0	0.0	1.0
GCF amount (μl/pocket)	Healthy sites	0.5 ± 0.3	0.2	0.3	0.6
	Diseased sites	1.3 ± 0.8^***^	0.6	1.1	1.7
PD (mm)	Healthy sites	2.1 ± 0.7	2.0	2.0	2.0
	Diseased sites	4.9 ± 1.1^***^	4.0	5.0	5.5
CAL (mm)	Healthy sites	3.1 ± 1.5	2.0	3.0	4.0
	Diseased sites	6.6 ± 2.1^***^	5.0	6.0	8.0
GI	Healthy sites	0.2 ± 0.4	0.0	0.0	0.0
	Diseased sites	1.3 ± 0.7^***^	1.0	1.0	2.0
ABR ratio (%)	Healthy sites	15.2 ± 19.0	0.0	9.2	27.9
	Diseased sites	47.0 ± 23.4^***^	28.8	44.1	66.4
ALP activity (μU/pocket)	Healthy sites	78.4 ± 129.6	19.3	30.8	80.5
	Diseased sites	445.7 ± 849.6^***^	39.0	127.5	386.1
Protein amount (μg/pocket)	Healthy sites	14.0 ± 10.3	6.1	11.6	19.1
	Diseased sites	33.0 ± 25.5^***^	16.1	25.3	39.0
Hb amount (ng/pocket)	Healthy sites	51.2 ± 48.8	7.4	35.7	86.8
	Diseased sites	86.5 ± 43.9^***^	53.6	96.6	119.7

Healthy sites PD ≤ 4 mm, BOP (−); *n* = 76

Diseased sites PD ≥ 4 mm, BOP (+); *n* = 76

^***^P < 0.001 versus healthy sites

### Correlation between Hb amount and other parameters in GCF

A significant correlation was observed between every parameter (PlI, GCF amount, GI, PD, CAL, BOP, ABR ratio, ALP activity, and protein amount) at 152 sites and Hb amount in GCF of the same site (*P* < 0.05) (Table 3).

**Table 3:** Correlation between Hb amount and clinical and biochemical parameters (*n* = 152 sites)

Clinical and biochemical parameters	Mean ± SD	Correlation coefficient	First quartile	Median	Third quartile
PlI	0.4 ± 0.6	0.178^*^	0.0	0.0	1.0
GCF amount (μl/pocket)	0.9 ± 0.7	0.399^***^	0.3	0.6	1.2
PD (mm)	3.5 ± 1.6	0.322^***^	2.0	4.0	5.0
CAL (mm)	4.9 ± 2.6	0.271^***^	3.0	4.0	6.5
GI	0.7 ± 0.8	0.365^***^	0.0	1.0	1.0
BOP	0.5 ± 0.5	0.368^***^	0.0	0.5	1.0
ABR ratio (%)	31.1 ± 26.6	0.265^***^	6.8	28.6	48.7
ALP activity (μU/pocket)	262.1 ± 633.1	0.407^***^	22.8	64.1	174.4
Protein amount (μg/pocket)	23.5 ± 21.6	0.529^***^	8.8	17.1	28.6

Hb amount = 68.9 ± 49.5 ng/pocket

^***^P < 0.001, ^*^P < 0.05

### Setting of the Hb cutoff value by PD and BOP

**Fig. 2:**
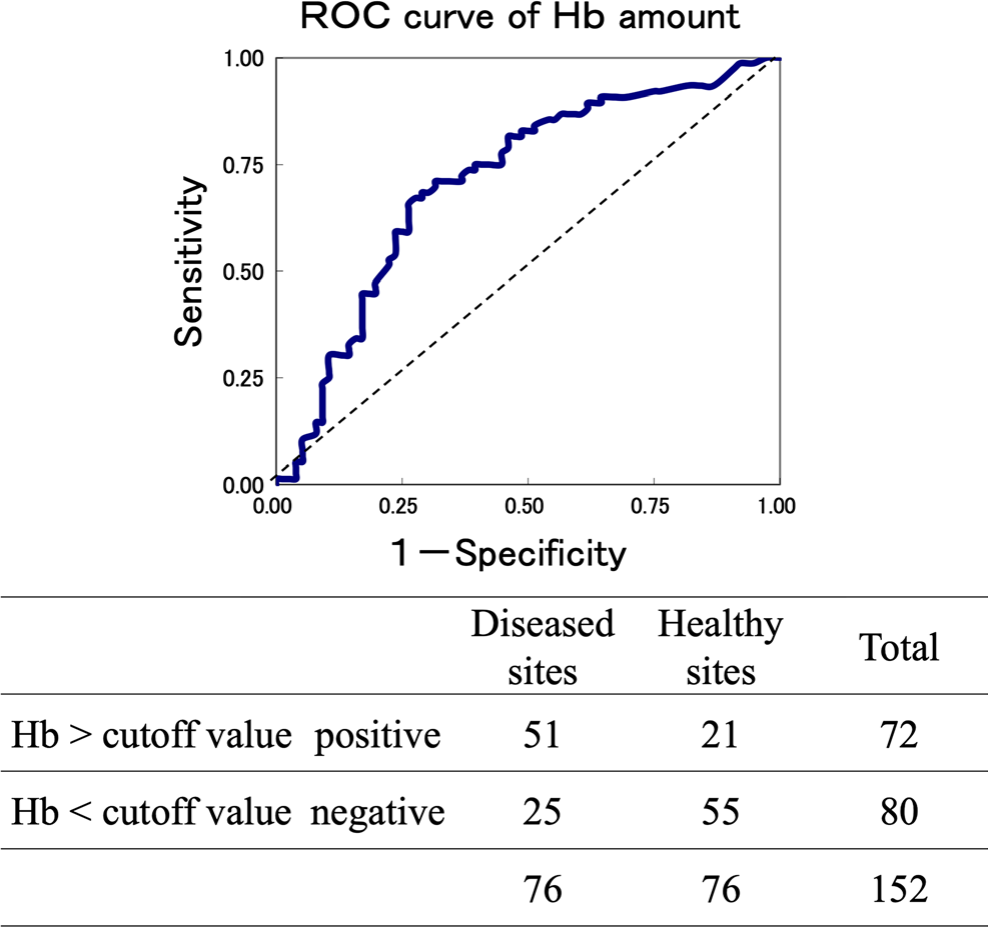
ROC curve and accuracy of the diagnosis

The Hb cutoff value was used to make the ROC curve (Fig. 2) and Youden index. The Hb cutoff value in healthy and diseased paired sites (*n* = 152) in the same oral cavity calculated by PD and BOP was 75.250 ng/pocket (Table 4).

**Table 4:** Cutoff value for Hb

Statistical indicators	Indicator value
Cutoff value	75.25 (ng/pocket)
Area under the curve	0.712
Sensitivity	0.671
Specificity	0.724
Positive predictive value	0.708
Negative predictive value	0.688
Proper diagnosis rate	0.697
Likelihood ratio	2.431

Analysis results using the ROC curve for healthy sites (defined as PD ≥ 4 mm, BOP (−); *n* = 76) and diseased sites (defined as PD ≤ 4 mm, BOP (+); *n* = 76)

### Comparison of parameters in each group reclassified using the Hb cutoff value

Clinical parameters (PlI, GCF amount, GI, PD, CAL, and ABR ratio) and biochemical parameters (ALP activity and protein amount) in healthy and diseased sites were reclassified by using the Hb cutoff value showing a high value with the area under the curve and correct diagnosis rate. We then created groups for less than the Hb cutoff value (L-Hb) and greater than the Hb cutoff value (H-Hb) in healthy and diseased sites (healthy L-Hb/diseased L-Hb groups and healthy H-Hb/diseased H-Hb groups, respectively). After reclassification of each test result into these four groups, Kruskal–Wallis analysis was carried out to compare parameters between each group, and the results revealed significant differences among groups for all clinical and biochemical parameters (*P* < 0.01). Furthermore, the Steel–Dwass test was performed for post hoc testing. A comparison between each parameter level included in the L-Hb group and that in the H-Hb group at each healthy or diseased site is shown in Table 5.

**Table 5:**
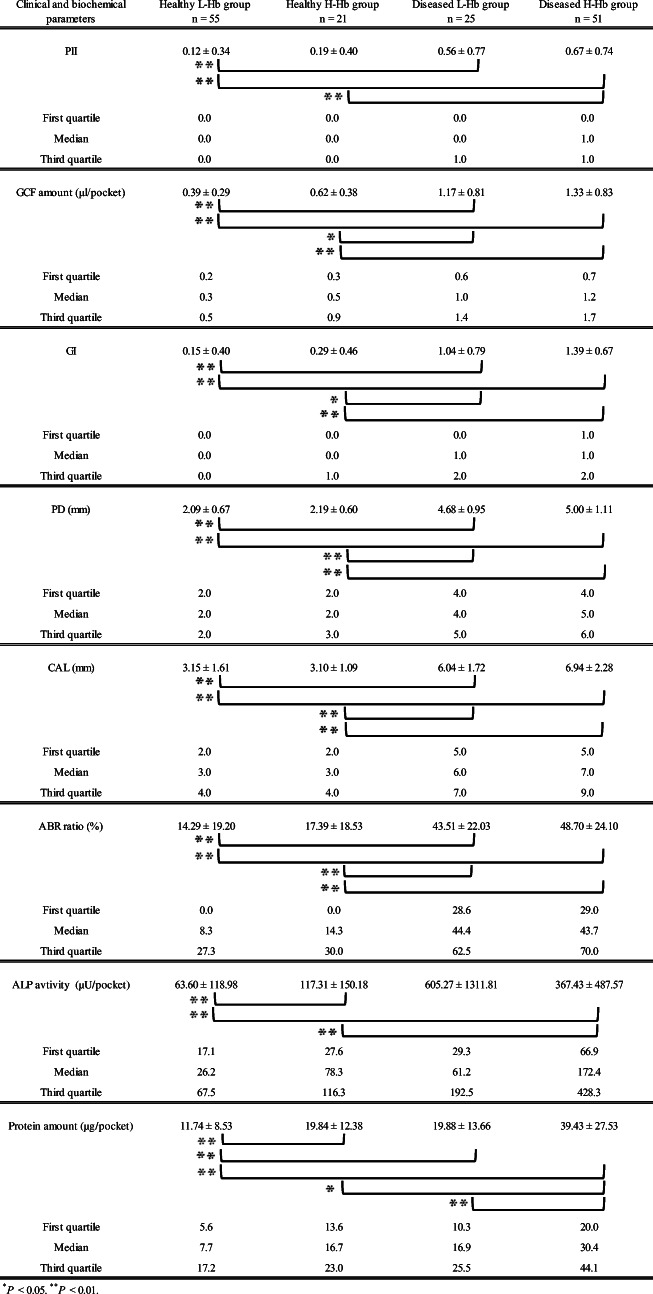
Comparison among healthy low-Hb cutoff group (healthy L-Hb group), healthy high-Hb cutoff group (healthy H-Hb group), diseased low-Hb cutoff group (diseased L-Hb group), and diseased high-Hb cutoff group (diseased H-Hb group)

At healthy sites, ALP activity and protein amount of the H-Hb group were significantly higher than those of the L-Hb group (*P* < 0.01). In contrast, at diseased sites, a significant difference between L-Hb and H-Hb groups was observed only for protein amount (*P* < 0.01).

However, there were no significant differences between L-Hb and H-Hb groups for PlI, GCF amount, GI, PD, CAL, and ABR ratio at healthy sites or PlI, GCF amount, GI, PD, CAL, ABR ratio, and ALP activity at diseased sites.

## Discussion

Current periodontal tissue examinations, i.e., measurement of PD, CAL, BOP, and ABR ratio (clinical parameters), indicate the degree of tissue damage caused by a history of periodontal disease. In particular, BOP is important for maintenance of the attachment position during the SPT phase. Lang et al. reported that although acquisition of BOP (−) was important for maintaining the attachment position, a case showing attachment loss was slightly observed even in the case of BOP (−) [7]. Now, many reports on GCF components have concluded that the biochemical marker showing a high correlation with periodontal tissue examinations is effective for the onset and progression of periodontal disease [8]. In contrast, we speculated that detection of weak tissue damage in BOP (−) periodontal tissue can indicate premature periodontal disease.

Periodontal disease progression can be estimated by not only the presence of oral bacteria such as *Porphyromonas gingivalis* and *Treponema denticola* in GCF and saliva but also biochemical parameters in combination with clinical parameters [29–32]. In particular, measurements of matrix metalloproteinases, ALP, alanine aminotransferase and, Hb amount as biochemical parameters in GCF have gained attention, and clinical application of these tests is being studied [8, 10, 15, 16, 31].

We previously reported that Hb was observed in > 60% of GCF of 176 BOP (−) sites in 229 gingival sulci of 184 nonsmokers who were under regular SPT [10]. This result suggested that the inspection of Hb derived from microbleeding in gingival sulci may serve as an index for preclinical diagnosis. In this study, the amount of Hb in GCF of 152 sites strongly correlated with all clinical parameters evaluated (PlI, GCF amount, GI, PD, CAL, BOP, and ABR ratio), as well as biochemical parameters (ALP activity and protein amount) (Table 3).

To examine whether periodontal disease progression can be accurately determined by Hb examination, healthy sites (PD ≤ 4 mm, BOP (−)) and diseased sites (PD ≥ 4 mm, BOP (+)) in the same oral cavity of SPT patients were reclassified into four groups using the Hb cutoff value. Then, clinical and biochemical parameters between these four groups were compared. All clinical and biochemical parameters in diseased sites were higher than those in healthy sites in the same oral cavity (Table 5). In contrast, ALP activity and protein amount, which increase with periodontitis, were significantly higher in the healthy H-Hb group (117.31 ± 150.18 μU/pocket and 19.84 ± 12.38 μg/pocket, respectively) than in the healthy L-Hb group (63.60 ± 118.98 μU/pocket and 11.74 ± 8.53 μg/pocket, respectively) (*P* < 0.01). These results indicated that slight tissue damage may have occurred even in healthy gingival sulcus as defined by BOP and PD measurements. Moreover, our findings suggest that Hb examination is a promising candidate marker in the initial condition of periodontal disease.

The method used for quantification of Hb in GCF in this study is based on the IC method using human Hb monoclonal antibody, and it can be carried out with high accuracy and short chairside time [10]. Therefore, Hb examination of GCF may capture periodontal disease in the initial state and become a powerful diagnostic tool for pre-symptomatic diagnosis. However, this investigation was a cross-sectional study and only mentioned the possibility of pre-symptomatic diagnosis. Therefore, it is necessary to verify not only the long-term prognosis but also the effects of past treatment history. In addition, these findings must be confirmed using a marker such as matrix metalloproteinase-8 [33] that is effective for the pre-symptomatic diagnosis of periodontal disease. We believe that the key for pre-symptomatic diagnosis is the detection of specific biochemical markers observed in GCF that fluctuate during BOP (−). In this study, during periodontal tissue stabilization showing BOP (−), ALP activity and protein amount indicating tissue damage were also significantly increased when Hb was significantly increased. It is urgently necessary to create a standard Hb value in GCF with high sensitivity and specificity to indicate periodontal tissue damage for pre-symptomatic diagnosis of periodontal disease.

## Conclusion

In this study, Hb examination in GCF detected slight tissue damage even in clinically periodontal healthy sites. These results suggest that Hb examination is a promising candidate marker for the pre-symptomatic state in periodontal disease.
